# Weakly supervised detection and classification of basal cell carcinoma using graph-transformer on whole slide images

**DOI:** 10.1038/s41598-023-33863-z

**Published:** 2023-05-09

**Authors:** Filmon Yacob, Jan Siarov, Kajsa Villiamsson, Juulia T. Suvilehto, Lisa Sjöblom, Magnus Kjellberg, Noora Neittaanmäki

**Affiliations:** 1 AI Sweden, Gothenburg, Sweden; 2 AI Competence Center, Sahlgrenska University Hospital, Gothenburg, Sweden; 3 Department of Laboratory Medicine, Institute of Biomedicine, Sahlgrenska Academy, University of Gothenburg, Gothenburg, Sweden

**Keywords:** Cancer, Computational biology and bioinformatics, Diseases, Health care, Medical research

## Abstract

The high incidence rates of basal cell carcinoma (BCC) cause a significant burden at pathology laboratories. The standard diagnostic process is time-consuming and prone to inter-pathologist variability. Despite the application of deep learning approaches in grading of other cancer types, there is limited literature on the application of vision transformers to BCC on whole slide images (WSIs). A total of 1832 WSIs from 479 BCCs, divided into training and validation (1435 WSIs from 369 BCCs) and testing (397 WSIs from 110 BCCs) sets, were weakly annotated into four aggressivity subtypes. We used a combination of a graph neural network and vision transformer to (1) detect the presence of tumor (two classes), (2) classify the tumor into low and high-risk subtypes (three classes), and (3) classify four aggressivity subtypes (five classes). Using an ensemble model comprised of the models from cross-validation, accuracies of 93.5%, 86.4%, and 72% were achieved on two, three, and five class classifications, respectively. These results show high accuracy in both tumor detection and grading of BCCs. The use of automated WSI analysis could increase workflow efficiency.

## Introduction

Basal cell carcinoma is the most common form of skin cancer in humans. The incidence is as high as the incidence of all other cancers combined^1^. Further, the number of BCC cases is increasing globally^2–4^. Although metastasis and death are rare, BCCs can cause significant morbidity due to aggressive and destructive local growth^5^.

BCCs are a heterogeneous group of tumors with different growth patterns. Internationally, BCCs are classified into two broad categories based on histopathologic features: low-risk and high-risk subtypes^6^. These categories can be further classified in subclasses. Swedish pathologists, for example, classify BCCs according to the “Sabbatsberg model” which includes three risk categories: (a) “low-aggressive” subtypes which are further divided into superficial (type Ib) and nodular (type Ia), and (b) “medium-aggressive” (type II) which includes less aggressive infiltrative subtypes that grow in a more well-defined manner and more superficially compared to the high-aggressive tumors and (c) “high-aggressive” (type III), more aggressive, infiltrative and morphea form subtypes^7^. The correct assessment of the subtype is crucial for planning the relevant treatment. However, there is a significant inter-pathologist variability when grading tumors^8^ and reporting the subtype^9,10^.

Moreover, given the time-consuming process of evaluating histological slides combined with an increasing number of samples delays diagnosis and increases costs^11^. To reduce diagnosis time and inter-observer variations, deep learning^12^ approaches have been actively investigated. Deep learning enables the implementation of computational image analysis in pathology, which provides the potential to increase classification accuracy and reduce interobserver variability^13,14^. Interestingly, even unknown morphological features associated with metastatic risk, disease-free survival, and prognosis may be revealed^15,16^.

In early research works computational histology methods required pixel-wise annotations, i.e., delineating specific regions on WSI by pathologists^17^. Using pixel-wise annotation, however, is time-consuming. Further, such approaches do not generalize to real-world data^18^. As an alternative, a weakly supervised learning framework has been a widely adopted method for WSI classification. The common technique within weakly supervised learning is multi-instance learning (MIL)^19^. This approach can use WSI-level labels, i.e., labels not associated with a specific region, without losing performance^20^. The technique treats the set of instances (patches of a WSI) as a bag. The mere instance of a positive case patch makes the bag (WSI), positive, otherwise, it is treated as negative. MIL requires that the WSI are partitioned into a set of patches, often without the need for data curation^18^.

The later works have increasingly added a self-supervised contrastive learning paradigm in extracting better feature vectors. In these paradigms pre-trained CNN models are tuned using a contrastive learning framework in a contained manner^21^. Adding these components into MIL approaches has proven to provide better performance^22,23^. However, the MIL framework fundamentally assumes the patches as independently and identically distributed, neglecting the correlation among the instances^19,24^. Neglecting the correlation affects the overall performance of the classification models. Instead, the spatial correlation can be captured using the graph neural networks, which in turn increases model performance^25–27^.

Recently, Transformers^28^ have made a great leap in the AI front by introducing the capability to incorporate context among a sequence of tokens in natural language processing tasks e.g. GPT-3^29^. Inspired by the success of transformers in natural language processing, Dosovitskiy et al.^30^ proposed Vision Transformer (ViT), a method for image classification tasks that takes patches of an image as input. This enables capturing the sequence of patches (tokens) and considers the position of images (context) using positional embeddings. Consideration of the positional relationship (contextual information) shows that ViT can perform better than CNN, especially when using features obtained from self-supervised contrastive models. In addition, vision transformers require substantially fewer data and compute resources relative to many CNN-based approaches^30,31^. Further, the relative resilience to noise, blur, artifacts, semantic changes, and out-of-distribution samples could contribute to better performance^32^.

In medical images, transformers have been applied in image classification, segmentation, detection, reconstruction, enhancement, and registration tasks^32^. Specifically, in histological images, vision transformers have been successfully applied to different histological images related tasks, including in the detection of breast cancer metastases, and in the classification of cancer subtypes of lung, kidney and colorectal cancer^33,34^. Given the success of vision transformers in many medical applications and the capability of graph neural networks to capture correlation among patches, we adopt the combination of graph neural networks and Transformers to detect and classify BCCs.

## Results

**Figure 1:**
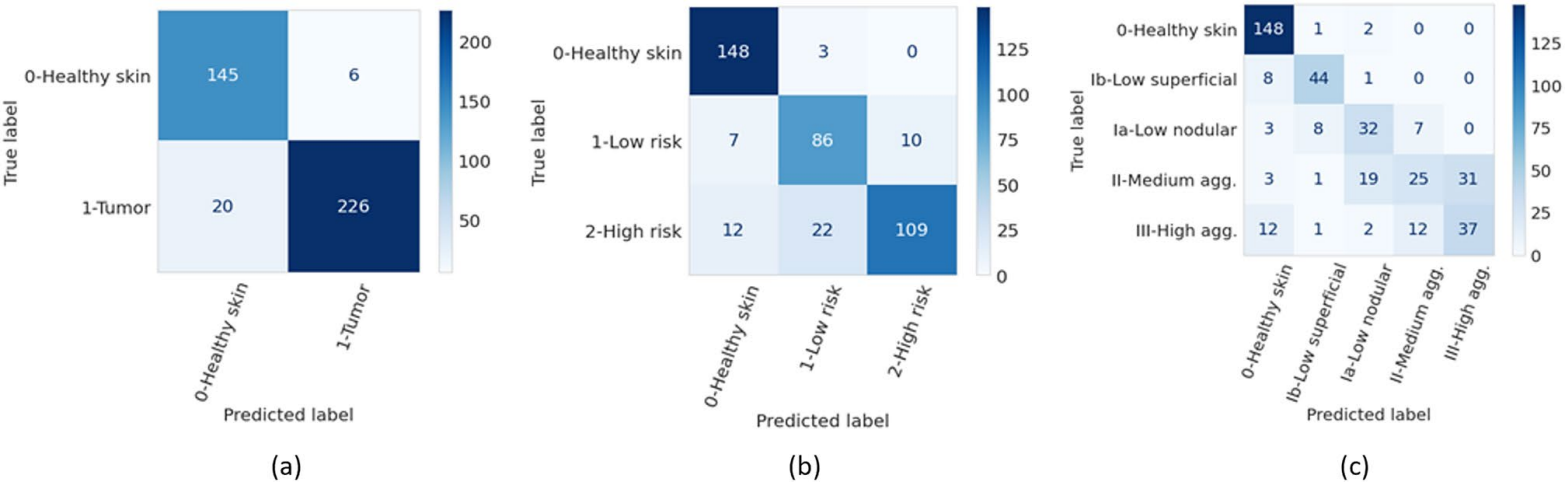
Confusion matrices of the ensemble models for the three different classification tasks (T) on the test set. (**a**) binary classification (T1, tumor or no tumor), (**b**) three class classification (T2, no tumor and two grades of tumor), (**c**) five class classification (T3, no tumor and four grades of tumor).

**Figure 2:**
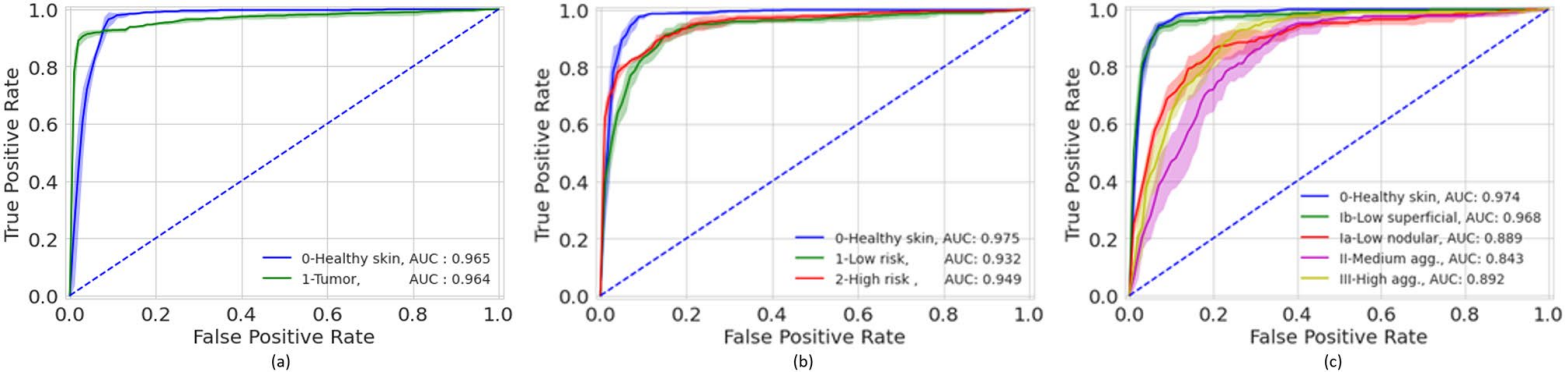
Mean ROC curves of the five-fold cross-validation models based on a test set for the different classification tasks (T). (**a**) binary classification (T1), (**b**) three class classification (T2), (**c**) five class classification (T3).

**Figure 3:**
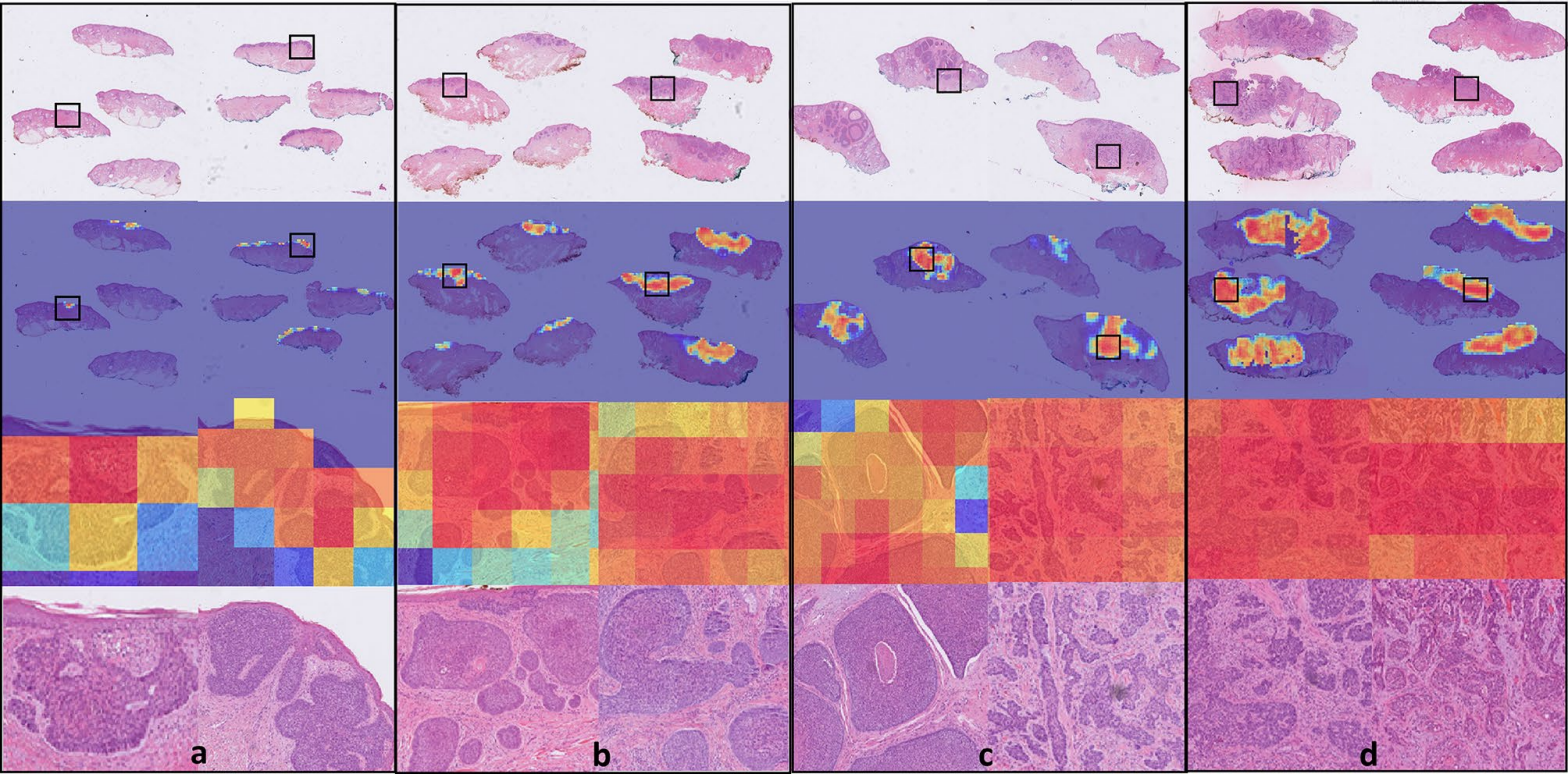
Visualization of class activation maps (rows 2 and 3) and corresponding H&E images (rows 1 and 4). The class activation maps are built for the binary classification task (no tumor, tumor) with the areas of tumor emphasized. Representative examples are shown for all four BCC grades: (**a**) superficial low aggressive, (**b**) nodular low aggressive, (**c**) medium aggressive, (**d**) high aggressive. Rows 3 and 4 represent close up images from the areas marked with black boxes. The slides have been cropped to focus on the tissue after running the model.

The accuracies of the ensembles comprised of the 5 graph-transformer models on the test set were 93.5%, 86.4%, and 72.0% for the two-class, three-class, and five-class classification tasks, respectively. Moreover, the sensitivity of detecting healthy skin and tumors reached 96% and 91.9%, respectively. The performance of the ensemble models on the test set is summarized in Table 1 and the associated confusion matrices are shown in Fig. 1. Figure 2 shows the average ROC curve of the separate cross-validation models against the test set. Heatmaps were used to visualize the regions of WSI that are highly associated with the label. Figure 3 shows tumor regions of different BCC subtypes that were correctly identified by a Graph-Transformer model.

**Table 1:** Model performance with 1, 2, and 4 BCC grades on the test set based on an ensemble model comprising of the 5 cross-validation model splits.

Tasks	Sub-class	Accuracy (%)	Sensitivity (%)	Specificity (%)
2 classes (Task 1)	0-Healthy skin	93.5	96.0	91.9
	1-Tumor		91.9	96.0
3 classes (Task 2)	0-Healthy skin	86.4	98.0	92.3
	1-Low risk		83.5	91.5
	2-High risk		76.2	96.1
5 classes (Task 3)	0-Healthy skin	72.0	98.0	89.4
	1-Superfial low		83.0	96.8
	2-Nodular low		64.0	93.1
	3-medium aggressive		31.6	94.0
	4-High aggressive		57.8	90.7

## Discussion

In this paper, we used a graph-transformer for the detection and classification of WSIs of extraction with BCC. The developed deep-learning method showed high accuracy in both tumor detection and grading. The use of automated image analysis could increase workflow efficiency. Given the high sensitivity in tumor detection, the model could assist pathologists in identifying the slides containing tumor and indicating the tumor regions on the slides and possibly reduce the time needed for the diagnostic process in daily practice. The use of high-accuracy automated tumor grading could further save time and potentially reduce inter- and intra-pathologists’ variability.

Our study is among the first to apply two and four grading of BCC on WSI using deep learning approaches. Our method reached high AUC values of 0.964–0.965, 0.932–0.975 and 0.843–0.976 on two, three (two grades), and five class (4 grades) classifications, respectively. Previously, Campanella et al.^18^ used a significantly larger dataset of totally 44,732 WSIs including 9,962 slides with wide range of neoplastic and non-neoplastic skin lesions of which 1,659 were BCCs. They achieved high accuracy in tumor detection and suggested that up to 75% of the slides could safely be removed from the workload of pathologists. Interestingly, Gao et al.^35^ compared WSIs and smartphone-captured microscopic ocular images of BCCs for tumor detection with high sensitivity and specificity for both approaches. However, no tumor grading was applied in these studies. To the best of our knowledge, there is no open-source dataset on grading of BCC. This makes it difficult to compare the results of this work against a baseline. One advantage of our study is that data is available as an open data set which will enable progress in this area.

In another study regarding BCC detection attention patterns of AI were compared to attention patterns of pathologists and observed that the neural networks distribute their attention over larger tissue areas integrating the connective tissue in its decision-making^36^. Our study used weakly supervised learning, where the labels were assigned on a slide level. This approach instead of focusing on small pixelwise annotated areas, gives the algorithm freedom to evaluate larger areas including the tumoral stroma. Furthermore, slide-wise annotation is significantly less time-consuming than pixel-wise annotations.

A limitation of our study is the somewhat limited size of the dataset. As the number of classes increases the performance reduced significantly. This could be attributed to a reduced number of WSI per class in the training set. For example, it was more difficult for the model to differentiate between the BCC subtype Ia and subtype Ib in 5 class classification tasks but relatively easier to differentiate the low and high aggressive classes in 3 class classification task, Fig. 2. With the availability of more data, the performance would most likely increase.

Even though this work didn’t make systematic inter-observer variability analysis, the two pathologists involved in the annotation of the dataset into 4 different grades (5-class classification) differed in 6.7% of WSIs. The annotation of those WSIs were corrected with consensus along with a third senior pathologist, which is not the case in real life situations. Using tools, such as the one proposed in this work, would likely reduce the inter-pathologist variability. More studies on the subject are warranted.

A limitation in our study is the imbalance in the dataset in different tasks. We included several (1–18 slides) per tumor. Each slide was classified individually. Even though we aimed to include as many WSIs in each tumor group there were differences between the groups. The more aggressive tumors were bigger and thus had more slides. Also the fact that within the same tumor several BCC subtypes were presented affected the number of the WSIs in each group. Since we included several slides from the same tumor not all slides showed tumor. Thus totally 744 included slides represented healthy skin as shown in Table 2. This caused imbalance in the dataset especially in the task 2 and 3 were the largest group was the healthy skin. Furthermore, the fact that a few BCC cases did not show any tumor slides could be because of some slides needed to be removed due to low quality in the scanning.

**Table 2:** The distribution of BCCs and WSIs into the different classes.

	All included		Training and validation set		Hold-out test set	
	Number of cases	WSIs	Number of cases	WSIs	Number of cases	WSIs
Total	479	1832	369	1435	110	397
2 classes (Task 1)						
No tumor	4	745*	2	594	2	151
Tumor	475	1087	367	841	108	246
3 classes (Task 2)						
No tumor	4	745	2	594	2	151
Low risk	219	506	178	403	41	103
High risk	256	581	189	438	67	143
5 classes (Task 3)						
No tumor	4	745	2	594	2	151
Low aggressive superficial	81	230	63	177	18	53
Low aggressive nodular	138	276	115	226	23	50
Medium aggressive	138	294	98	215	40	79
High aggressive	118	287	91	223	27	64

BCCs were classified according to the WSI with the worst grade tumor belonging to that BCC.

*These represent slides from BCC excisions which represented healthy skin.

Furthermore, many of the WSIs had composite subtypes and these sometimes were present on the same slide. Such cases are typical in BCC to have an admix from multiple types, i.e., cases with more than one pathologic pattern^37^. The proportion of mixed histology cases can reach up to 43% of all cases^38^. Up to 70% of mixed BCC cases can contain one or more aggressive subtypes^39^. Despite such characteristics of mixed pattern per WSI, our models were able to detect the worst BCC subtype per slide with an accuracy of 86.4% in the three-class classification, and 72.0% in the five-class classification tasks, as shown in Table 1.

Further, each slide had pen marks that indicate extraction index (corresponding to extraction id) in which some cases can be as large as the tissue on the WSI. Since the dataset is split based on a patient index, the pen marks in the training set are different from that of test set, and the model is not affected by the similarities of the handwritten characters. The pen marks were not identified as tissue by the tiler and were therefore not included in the training patches. Moreover, the WSIs had different colors and artifacts, slice edges, inconsistencies, scattered small tissues, spots, and holes. Despite these variations among the WSIs, the models treated handwritten characters as background and other variations as noise.

This work, to the best of our knowledge, is the first approach that uses transformers in the grading of BCC on WSI. The results show high accuracy in both tumor detection and grading of BCCs. Successful deployment of such approaches could likely increase the efficiency and robustness of histological diagnostic processes.

## Methods

### Dataset

The dataset was retrospectively collected at Sahlgrenska University Hospital, Gothenburg, Sweden from the time period 2019–2020. The complete dataset contains 1831 labeled WSI from 479 BCC excisions (1 to 18 glass slides per tumor), Table 2. The slides were scanned using a scanner NanoZoomer S360 Hamamatsu at 40X magnification. The slide labels were then removed using an open-source package called anonymize-slide^40^.

The dimensions of the WSIs ranged from 71,424 to 207,360 px, with sizes ranging 1.1 GB to 5.3 GB (in total 5.6 TB). Moreover, almost all samples had multiple sectioning levels per glass slide. Before scanning, the glass slides were marked with letter ‘B’ and up to 3 digits indicating which slides represented the same tumor.

The scanned slides were then annotated at WSI-level into 5 classes (no-tumor and 4 grades of BCC tumor), in accordance with the Swedish classification system. When several growth patterns of tumors were detected, the WSIs were classified according to the worst possible subtype. The annotations were performed by two pathologists separately. In the cases where the two main annotators had differing opinions (6.7% of WSIs), a third senior pathologist was brought in, and a final annotation decision was made as a consensus between the three pathologists.

**Figure 4:**
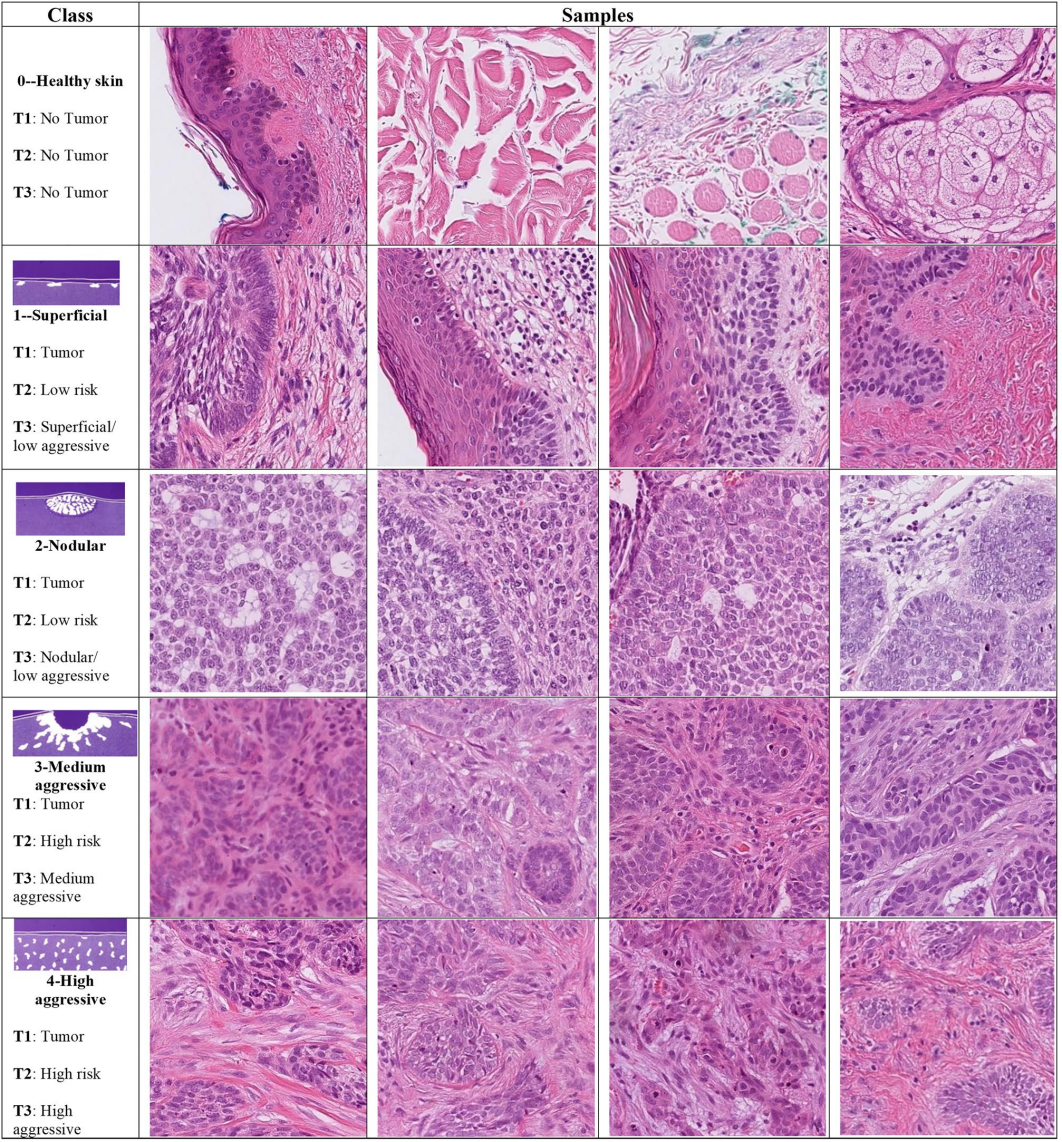
Samples of BCC subtypes used in the three classification tasks (T): **T1** (tumor or no tumor), **T2** (no tumor and two grades of tumor), and **T3** (no tumor and four grades of tumor), arranged by a pathologist in accordance with “Sabbatsberg model”^7^. Depending on the classification task at hand, the samples in each row are assigned a different grade of tumor.

The dataset was set out for use for 3 classification tasks. The first task (T1) was detecting the presence of tumors through binary classification (tumor or no tumor). The second task (T2) was classified into three classes (no tumor, low-risk and high-risk tumor, in line with WHO grading systems). The third task (T3) was classing the dataset into 5 classes (no tumor, and 4 grades of BCC; low aggressive superficial, low aggressive nodular, medium aggressive and highly aggressive, in line with the Swedish classification system). In the two-grade classification tasks, the labels were converted to cases of low aggressive (Ia and Ib) and high aggressive (II and III). Figure 4 shows patches of BCCs and their corresponding classes in the three classification tasks (indicated as T1, T2, and T3).

### Feature extraction

**Figure 5:**
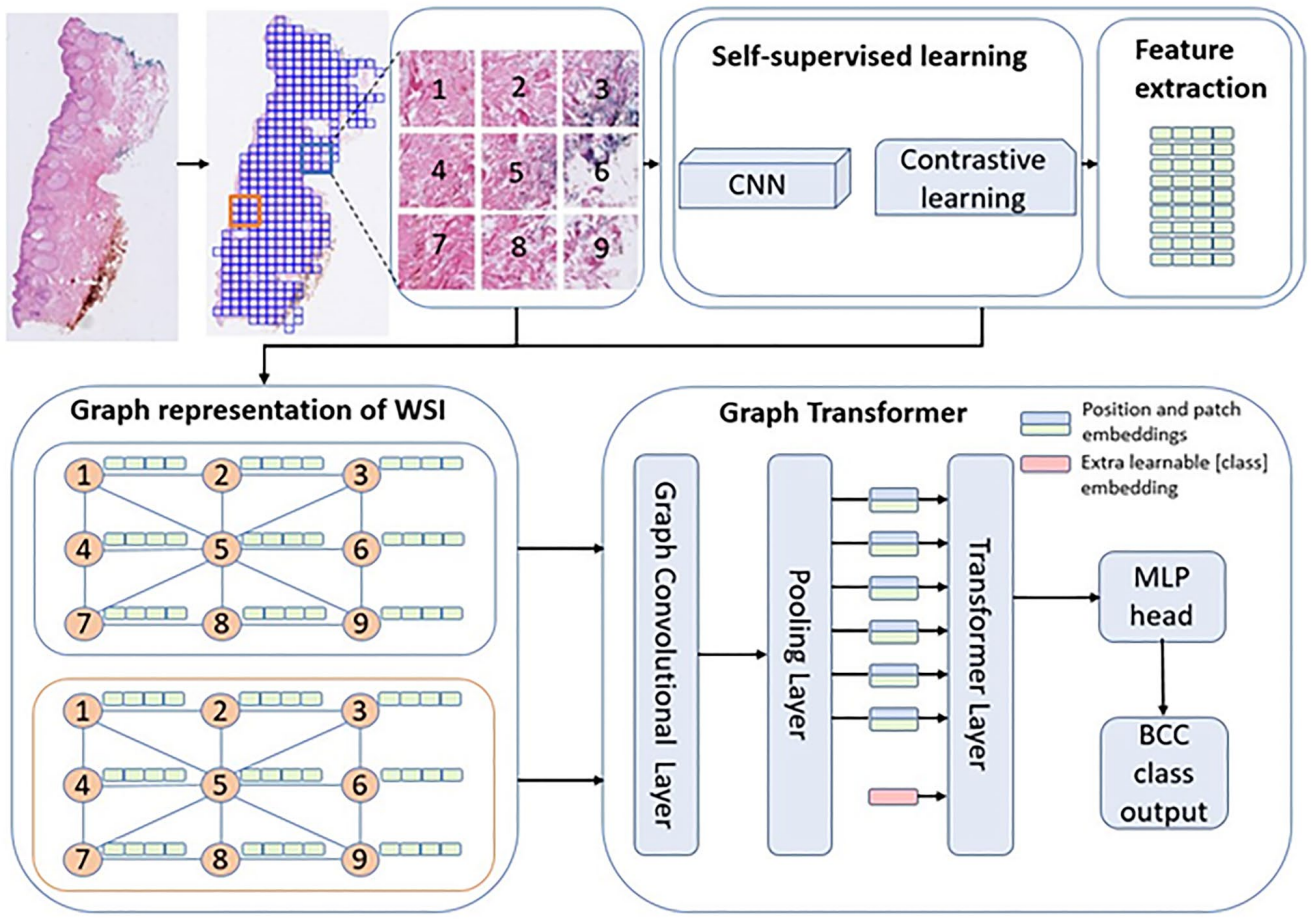
Method overview (adapted from Zheng et al.^27^). The WSI is first tiled into patches and feature extracted via self-supervised learning. The extracted features become the nodes of a graph network, which become the inputs to a graph-transformer classifier.

An overview of the method is shown in Fig. 5. Given that WSIs were large, conventional machine-learning models could not ingest them directly. Hence the WSIs were first tiled into patches. The WSIs were tiled into 224 by 224 patches at 10X magnification with no overlap using OpenSlide^41^. The patches with at least 15% tissue areas were kept while others were discarded. The number of patches ranged from 22–14,710 patches per WSI. In total, 5.2 million patches were generated for the training set. As stated above, there was variability among the WSIs including color differences, artifacts, etc. Despite the differences among patches, no image processing was made before or after tiling.

Once the patches were tiled, features were extracted using a self-supervised learning framework, SimCLR^21^. Using a contrastive learning approach, the data was augmented, and sub-images were then used to generate a generic representation of a dataset. The algorithm then reduced the distance between the same image and increased the distance between different images (negative pairs)^21^. In this step, using Resnet18 as a backbone and all patches as a training set, except the patches from the hold-out test set, a feature vector for each patch was extracted. For training SimCLR, Adam optimizer with weight decay of 10^–6^, and batch size of 512 and 32 epochs were used. The initial learning rate 10^–4^ was scheduled using cosine annealing.

### Graph convolutional network construction

The features generated from self-supervised contrastive learning were used to construct the graph neural networks. Using contrastive learning feature vectors of each patch were extracted. Since each patch is connected to the nearest neighbor patch by its edges and corners, tiling breaks the correlation among the patches. The correlation among patches is typically captured via positional embeddings^30^. Since histological patches are spatially correlated in a 2D space, the positional embeddings could better be captured via a graph network^27^.

A patch is connected to a neighboring patch by 4 sides and 4 corners, hence in total 8 edges. A set of 8-node adjacency matrices was used to create a graph representation of a WSI. Then the positional embedding captured via the adjacency matrix is used to construct a graph convolutional network. The feature vectors of the patches became the nodes of the graphs.

**Figure 6:**
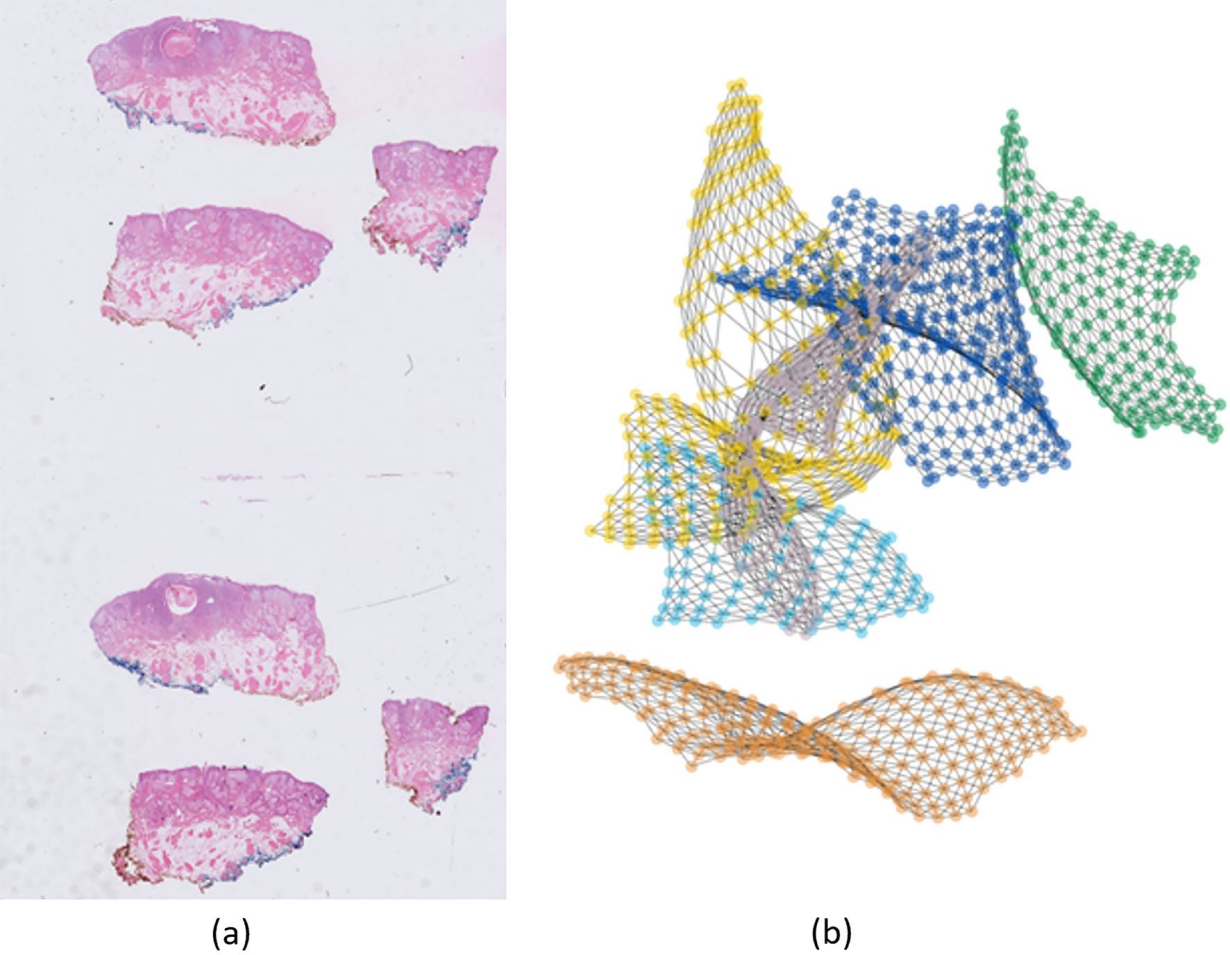
An example of a WSI and its graph network. (**a**) WSI with six tissue sections, (**b**) six disconnected components of a graph network. The disconnected components are randomly placed in space. Each node represents a patch (patches not shown in the figure for better visualization).

Zheng et al.^27^ showed results of using a fully connected graph, that is, a single tissue per slide. In this work, we show that the same approach works with a disconnected graph representing multiple tissues per WSI. It is worth noting that almost all WSIs in our dataset had multiple tissues per slide, i.e., there were no correlations among the separate tissues due to non-tissue regions. This results in a disconnected graph as shown in Fig. 6. It is worth noting that the distance between the components of the disconnected graph as well as their position in space has no effect on the performance of the model.

### Vision transformer

Once the graph convolutional network was built, the network was fed to a ViT. Generally, the transformer applies an attention mechanism that mimics the way humans extract important information from a specific image or text, ignoring the information surrounding the image or text^42^. Self-attention^28^ introduced a function that uses queries, keys, and values vectors, mapped from the input features. Using these vectors, it applies multiple-head self-attention to extract refined features, allowing it to understand the image as a whole rather than just focusing on individual parts. Further, the self-attention function is accompanied by a multilayer perceptron (MLP) block which is used in determining classes. In this work, we used the standard ViT encoder architecture along with graph convolutional network for classification of BCC subtypes.

Moreover, the computational cost of training ViT can be high depending on the input size. The number of patches can be large depending on the size of images and tissue size relative to the WSI. This resulted in a large number of nodes, which were computationally hard to be applied directly as input to the transformer. To reduce the number of nodes to the extent that the ViT can digest the inputs, a pooling layer was added.

### Training the graph-transformer

There were 369 extractions (1435 WSIs) in the combined training and validation set. An additional dataset of 110 extractions (397 WSIs) were scanned separately to comprise a hold-out test set. The test set was handled separately and was held out from both SimCLR and graph-transformer models.

For training and validation, all slides relating to a specific extraction were always placed in the same set to avoid data leakage from similar slides. This necessitated dividing the dataset on the extraction level, resulting in uneven splits for cross-validation. Hence, a fivefold cross-validation was used for training. The outputs of the 5 models from the cross-validation folds were combined into one ensemble model through majority vote to provide final predictions against the test set. This step was performed for the two-, three-, and five-class classification tasks separately, Supplementary Table S1.

In training the models, the same hyperparameters were used for all the tasks. The models were configured with MLP size of 128, 3 self-attention blocks, and trained with batch size 4, 100 epochs and Adam optimizer’s weight decay 10^–5^, learning rate 10^–3^ with decay at steps 40 and 80 by 10^–1^. The training was performed on 2 GPUs on DGX A100. The training of SimCLR model took around 3 days. The training for graph transformers took around 25 min on average to converge. For a given WSI in the test set, from tiling to inference, took around 30 s.

### Visualization

To visualize and interpret the predicted results, a graph-based class activation mapping^27^ was used. The method computed the class activation map from the class label to a graph representation of the WSI by utilizing precomputed transformer and graph relevance maps. Using the method, heatmaps were overlayed on regions of the WSI associated with the WSI label.

### Supplementary Information

Supplementary Information.

## Data Availability

The datasets generated and/or analysed during the current study are available at https://doi.org/10.23698/aida/bccc.
