# Author Correction: Weakly supervised detection and classification of basal cell carcinoma using graph-transformer on whole slide images

**DOI:** 10.1038/s41598-023-40875-2

**Published:** 2023-08-22

**Authors:** Filmon Yacob, Jan Siarov, Kajsa Villiamsson, Juulia T. Suvilehto, Lisa Sjöblom, Magnus Kjellberg, Noora Neittaanmäki

**Affiliations:** 1 AI Sweden, Gothenburg, Sweden; 2 AI Competence Center, Sahlgrenska University Hospital, Gothenburg, Sweden; 3 Department of Laboratory Medicine, Institute of Biomedicine, Sahlgrenska Academy, University of Gothenburg, Gothenburg, Sweden

Correction to: *Scientific Reports* https://doi.org/10.1038/s41598-023-33863-z, published online 09 May 2023

The original version of this Article contained errors in Table 2, where values were missing under the column “WSIs”. The correct and incorrect columns appear below.

Incorrect:

**Table:** 

All included		Training and validation set	
Number of cases	WSIs	Number of Cases	WSIs
479	1832	369	1435
4	744*	2	593
475	1087	367	842
4	744	2	593
219		178	
256		189	
4	744	2	594
81		63	177
138		115	226
138		98	215
118		91	223

Correct:

**Table:** 

All included		Training and validation set	
Number of cases	WSIs	Number of cases	WSIs
479	1832	369	1435
4	745*	2	594
475	1087	367	841
4	745	2	594
219	506	178	403
256	581	189	438
4	745	2	594
81	230	63	177
138	276	115	226
138	294	98	215
118	287	91	223

The original Article has been corrected.

