# Supplementary material for: Weakly supervised detection and classification of basal cell carcinoma using graph-transformer on whole slide images
Source: Sci Rep. 2023 May 9;13:7555. doi: 10.1038/s41598-023-33863-z (PMC10169852; doi:10.1038/s41598-023-33863-z)
Supplement: Supplementary file 1 — Supplementary Information. [file 41598_2023_33863_MOESM1_ESM.docx]

**Supplementary materials:**

*Table S1* shows the average number of WSIs and splits into training, validation, and test set.

**Table S1.** The number of WSI per class in the test set and the different cross-validation folds for training and validation.

| **Tasks** | **Sub-class** | **Training** | **Validation** | **Testing** |
| --- | --- | --- | --- | --- |
| 2 classes  (Task 1) | 0-No tumor | 475.2 +- 7.2 | 118.8 +- 7.2 | 151 |
|  | 1-Tumor | 672.8 +- 30.1 | 168.2 +- 30.1 | 246 |
| 3 classes  (Task 2) | 0-No tumor | 474.4 +- 8.3 | 118.6 +- 8.3 | 151 |
|  | 1-Low risk | 322.4 +- 16.1 | 80.6 +- 16.1 | 103 |
|  | 2-High risk | 350.4 +- 17.3 | 87.6 +- 17.3 | 143 |
| 5 classes  (Task 3) | 0-No tumor | 474.4 +- 8.3 | 118.6 +- 8.3 | 151 |
|  | 1-Low aggressive, superficial | 140.8 +- 9.8 | 35.2 +- 9.8 | 53 |
|  | 2-Low aggressive, nodular | 181.6 +- 8.0 | 45.4 +- 8.0 | 50 |
|  | 3-Medium aggressive | 170.4 +- 3.8 | 42.6 +- 3.8 | 79 |
|  | 4-High aggressive | 180.0 +- 18.9 | 45.0 +- 18.9 | 64 |
